# The top 100 most-cited studies on monkeypox: a brief bibliometric analysis

**DOI:** 10.1097/MS9.0000000000001367

**Published:** 2023-10-02

**Authors:** Li Zheng, Yuan Yuan, Kaihua Zhang, Yan Wang, Xianjun Min

**Affiliations:** Departments of a Pharmacy; b Thoracic Surgery, China Aerospace Science and Industry Corporation 731 Hospital; c Department of Cardiovascular Medicine, Beijing Hospital, National Center of Gerontology, Institute of Geriatric Medicine, Chinese Academy of Medical Sciences, Beijing; d Department of Dermatology, Gansu Provincial Central Hospital; e Department of Dermatology, Gansu Provincial Maternity and Child-Care Hospital, Lanzhou, China

**Keywords:** bibliometric analysis, management, monkeypox, recognition, top 100 most cited

## Abstract

**Background:**

A plethora of monkeypox papers have been published; however, pinpointing key and pivotal studies can be challenging amongst the ever-expanding literature. Bibliometric analyses are helpful in identifying the most influential articles and their impact pertinent to this field, which has helped mould the recognition and management of monkeypox.

**Methods:**

The Web of Science Core Collection (WoSCC) was searched on 27 October 2022. The top 100 most-cited articles on monkeypox were identified and evaluated by author, country, institution, type of articles, theme, journal of publication, keywords, and citations.

**Results:**

The top 100 most-cited studies were published between 1997 and 2022, and the 5-year period with the largest number of articles was 2007–2011. The median number of citations among the top 100 most-cited articles was 78.23. Of the top 100 most-cited studies, 91 were original articles, and nine were reviews, the median of annual citations was significantly higher in the review group than the original group, 7.86 (4.20–13.80) versus 4.50 (3.07–7.59; *P*=0.023). The 100 articles were classified into different research theme, with the top three being Immunology (31%), Infectious Diseases (30%), and Virology (26%), respectively. The keywords with the highest co-occurrence frequency were “monkeypox”, “smallpox,” and “smallpox virus.” The largest number of articles in the top 100 were published in *Emerging Infectious Diseases* (*n*=13), followed by *Journal of Virology* (*n*=11), *Journal of Infectious Diseases* (*n*=5), and *PLoS One* (*n*=5). The authors identified 711 different authors from 195 institutions and 28 countries in the top 100 most-cited articles, with the majority based in the USA.

**Conclusion:**

The top 100 most-cited studies provide an important insight into the historical developments of monkeypox. The authors should strengthen the recognition and management of monkeypox worldwide and strengthen research cooperation among scholars in order to better respond to the ongoing or future outbreak.

## Introduction

Highlights

- Our study first provided a detailed bibliometric analysis of the top 100 most-cited articles in monkeypox, which may pave the way for further research.
- Our research pointed out the scholars, institutions, and countries that have made great contributions to the field of monkeypox, so as to facilitate communication between researchers around the world.
- Our research provides global scholars with the *status quo* and research trend of monkeypox research.

From May to July 2022, more than 10 000 monkeypox cases have occurred in more than 50 countries^1^, which has far exceeded the “worst situation” predicted by the model in the 1980s^2^. This shows that the monkeypox virus (MPXV) can be widely spread among people, which may become a serious threat to public health and cause global consequences.

MPXV is one of many zoonosis viruses belonging to the Orthopoxviral genus of *Poxviridae* ^3–5^. It was first identified and isolated in 1958 by Magnus *et al.* ^6^ from monkeys that got a pox-like disease at a research facility in Denmark, so it was named “monkeypox”^7^. The first human case was discovered in 1970, and the virus was isolated from a 9-month-old boy in the Democratic Republic of the Congo^8^. Since then, most human cases of monkeypox have been reported in the Democratic Republic of the Congo, Central Africa, and West Africa, and have gradually increased^9^.

The mortality rate of monkeypox was lower than that of smallpox, and the case fatality rate was about 10%^10^. In the 1970s, after the global eradication of smallpox, monkeypox cases began to receive global attention. But monkeypox did not really attract global attention until the outbreak of the epidemic in the USA in 2003. With the increasing number of monkeypox cases, the disease has attracted more attention from researchers^11^. Up to now, a plethora of monkeypox papers have been published; however, identifying key and pivotal studies can be challenging among the ever-expanding literature.

The influence of a published article is usually measured by the number of times that the article is cited^12^. If the study is considered to be very influential and important, it will usually be cited by other scholars in the field and get a great number of citations^13,14^. Therefore, identifying and analyzing the literatures that has been cited in large numbers in a certain field can provide researchers with important information in this field^15^. A citation analysis, also known as a bibliometrics analysis, is a method based on mathematics, statistics, and other disciplines to quantitatively summarize multiple pieces of information in a certain field^16^, which can provide research trends, research hotspots, and the overall development of the field^17,18^.

Based on the above-mentioned information, we conducted a citation analysis of monkeypox. In view of this, we used this method to identify and analyze the most-cited studies related to monkeypox so far, aimed to help mould our understanding and management of monkeypox and pave the way for further investigations in this field.

## Methods

We searched the records in the Web of Science Core Collection (WoSCC) database from database inception to 27 October 2022, with the following strategy: TS (Theme words)=monkeypox. In the end, we obtained all the documents related to the topic of monkeypox. Then, we limited the publication types to “ARTICLE” and “REVIEW,” restricted the published language to English, and ranked them by citation number. One reviewer (K.H.Z.) assessed whether it was a monkeypox study one by one until 100 studies were included, and another reviewer (L.Z.) checked the assessment results. At last, we included the top 100 English language studies and excluded papers that were not related to monkeypox or were case reports, books, letters, or other nonjournal publications. The full texts of each of these articles were downloaded. The final top 100 most-cited studies were included under the verification of infectious disease expert.

We used the visualization software of VOSviewer (1.6.16)^19^, Bibliometrix R package^20^, Citespace (6.2.1)^21^, and Excel (2019) for statistical analysis and draw map. The analysis included the general characteristics (e.g. authors, countries, and journals), the study topic (keywords), and the citations.

## Results

A total of 2011 research papers were retrieved from the WoSCC. The top 100 most-cited articles were listed in Table S1, Supplemental Digital Content 1, http://links.lww.com/MS9/A275 and ranked by citation number. The mean citations for a manuscript was 78.2, ranging from 382 to 31.

## Characteristics of the top most 100 cited studies

### Year of publication

**Figure 1:**
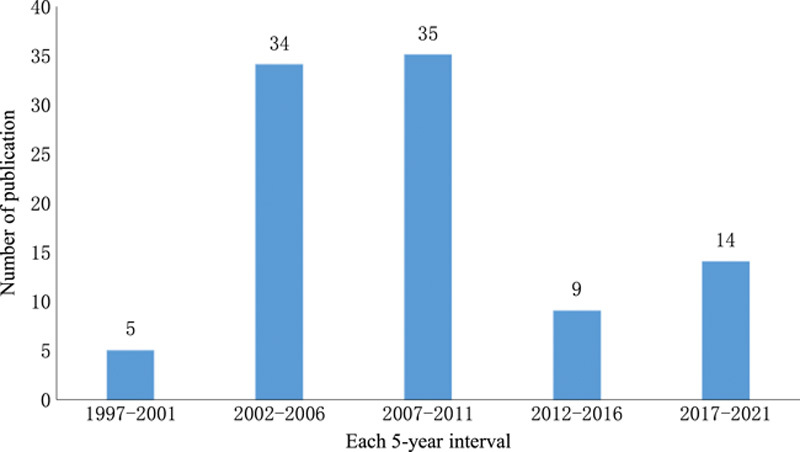
The number of 100 top-cited articles in monkeypox by each 5-year interval.

The top 100 most-cited studies were published between 1997 and 2022. The oldest literature (*n*=1) among the top 100 most-cited articles on the monkeypox was published in 1997, while the latest (*n*=3) were published in 2022 (Fig. 1). On dividing the list by each 5-year interval of publication, the largest number of articles were published in 2007–2011 (*n*=35), followed by 2002–2006 (*n*=34). Due to the incomplete 5-year period from 2022 to 2026, data for 2022 and beyond were not presented in Fig. 1.

### Type of studies

Of the top 100 most-cited studies, 91 were original articles (including 41 human experiments, 39 animal experiments, eight cell experiments, and three chemical experiments), and nine were reviews (Table 1). Among them, the mean citations per study in the original article was 77.8 (total=7083), and the mean citations per study in the review was 82.1 (total=739). The median of annual citations was significantly higher in the review group, 7.86 (4.20–13.80) versus 4.50 (3.07–7.59; *P*=0.023) [The quantitative data were compared using the Mann–Whitney *U*-test after being tested for normality by the Kolmogorov–Smirnov test, and Pearson’s correlation was used to assess the bivariate correlations (*P*<0.05 was considered statistically significant)].

**Table 1:** Type of the top 100 most-cited monkeypox studies

Type of study	Number of studies	Total citations	Mean citations per study	*H*-index
Original article				
Human experiment	41	7083	77.8	51
Animal experiment	39			
Cell experiment	8			
Chemical experiment	3			
Review	9	739	82.1	9

### Author, country, institution

The top 100 most-cited studies were produced by 711 authors whom from 195 institutions and 28 countries. Damon (*n*=27) produced the most studies, followed by Karem and Reynolds (Table S2, Supplemental Digital Content 1, http://links.lww.com/MS9/A275). Most of the 100 top-cited studies were from the USA (*n*=79), followed by Switzerland (*n*=12), Germany (*n*=8), England (*n*=8), and Congo (*n*=8; Table S3, Supplemental Digital Content 1, http://links.lww.com/MS9/A275). Ctr Dis Control & Prevent (Centers for Disease Control and Prevention) produced the most studies (*n*=30), followed by University of St Augustine (*n*=19), NIAID (National Institute of Allergy and Infectious Diseases, *n*=12; Table S4, Supplemental Digital Content 1, http://links.lww.com/MS9/A275).

In order to explore the cooperation of authors, countries, and institutions, we constructed a network visualization map by VOSviewer. The top 50 authors had stronger collaborative, and the top three scholars were Damon, Karem, and Olson (Fig. S1, Supplemental Digital Content 1, http://links.lww.com/MS9/A275). The top three collaborations countries were the USA, Switzerland and Germany (Fig. S2, Supplemental Digital Content 1, http://links.lww.com/MS9/A275), and the top three collaborations institutions were Ctr Dis Control & Prevent, University of St Augustine and NIAID (Fig. S3, Supplemental Digital Content 1, http://links.lww.com/MS9/A275).

### Journal of publication

The top 100 most-cited studies were published in 44 journals. *Emerging Infectious Diseases* published the most top-cited studies (*n*=13), followed by *Journal of Virology* (*n*=11), *Journal of Infectious Diseases* (*n*=5), and *PLoS One* (*n*=5). All journals were listed in Table S5, Supplemental Digital Content 1, http://links.lww.com/MS9/A275 with their impact factors. The results of the number of studies published within different impact factors (IF_2022_) range were shown in Figure S4, Supplemental Digital Content 1, http://links.lww.com/MS9/A275. Among them, nine studies were published in journals with IF_2022_ greater than 50.

## Research focus

### Web of science categories

The top 100 most-cited studies were classified into different categories. Table 2 showed the contains categories with five or more studies. The maximum number (*n*=31) of articles were about Immunology (*n*=31), followed by articles related to Infectious Diseases (*n*=30), Virology (*n*=26), and Microbiology (*n*=18).

**Table 2:** Web of science categories for the top 100 most-cited monkeypox studies

Rank	Web of science categories	Number of studies	Total citations	Mean citations per study	*H*-index
1	Immunology	31	1966	63.4	31
2	Infectious diseases	30	2105	70.1	30
3	Virology	26	1848	71.1	26
4	Microbiology	18	1314	73.0	18
5	Multidisciplinary sciences	9	884	98.2	9
6	Public environmental occupational health	7	467	66.7	7
7	Medicine research experimental	6	555	92.5	6
8	Pharmacology pharmacy	6	563	93.8	6
9	Biochemistry molecular biology	5	490	98.0	5
10	Tropical medicine	5	342	68.4	5

### Keywords

**Figure 2:**
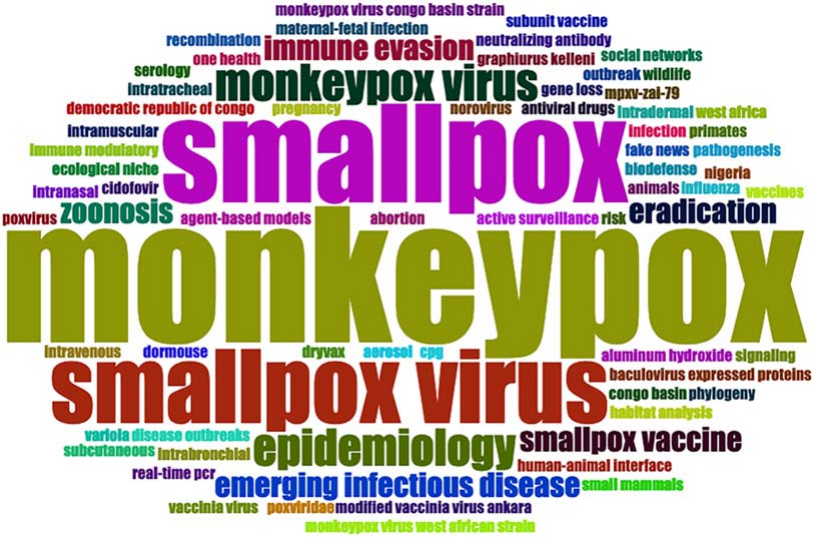
The wordcloud map of the author’s keywords.

After merging keywords with the same meaning, a total of 76 “author’s keywords” were included in the 100 top most-cited studies. The keywords with the highest co-occurrence frequency were “monkeypox,” “smallpox,” “smallpox virus,” “monkeypox virus,” “epidemiology,” “immune evasion,” and “eradication,” etc. (Fig. 2).

**Figure 3:**
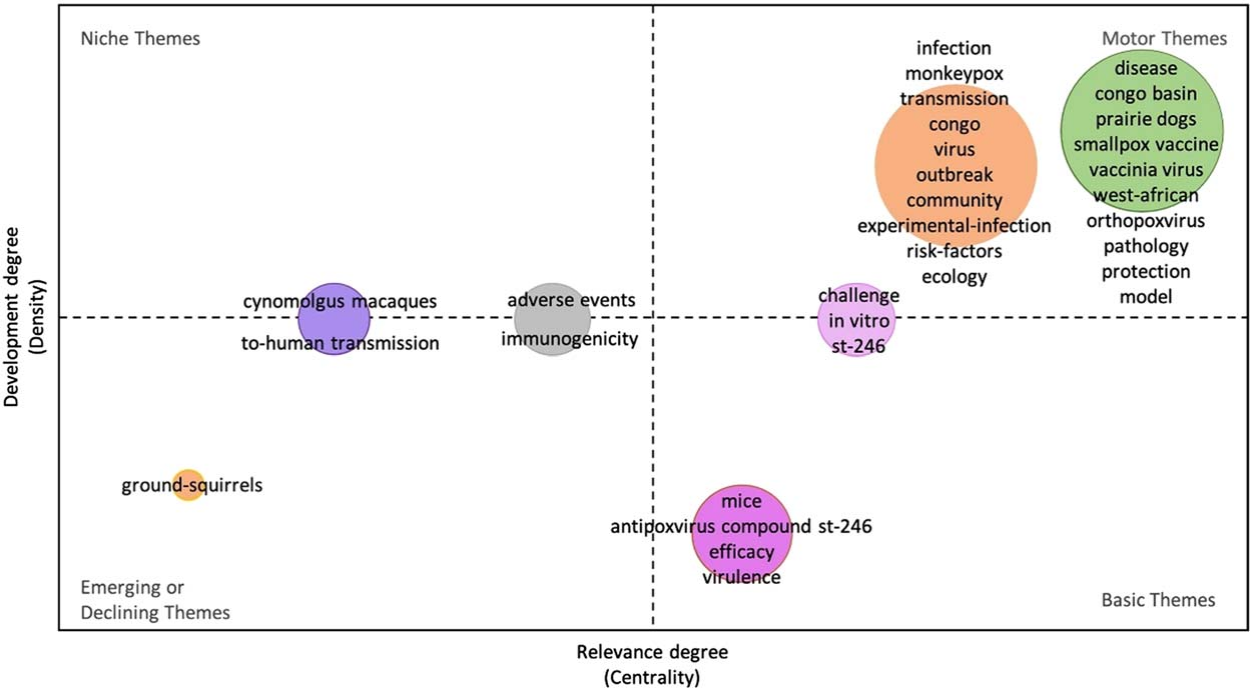
The thematic map basic on keywords (the upper right corner is the first quadrant, the upper left corner is the second quadrant, the lower left corner is the third quadrant, and the lower right corner is the fourth quadrant).

We presented the research trends by using the strategic diagram (Fig. 3), which highlighting four different kinds of topics and themes depending on the quadrant. The first quadrant is motor themes (higher values of centrality and density), which are well-developed and important themes for the structuring of a research field. The results found that the motor themes included the most keywords, and the representative keywords are “Congo,” “Community,” “Transmission,” “Outbreak,” “Risk-factors,” “Smallpox vaccine,” “West-African,” “Pathology,” and “protection,” etc. The second quadrant is basic themes (higher values of centrality and lower values of density), significant for the domain, and cross-cutting to its different areas. In this quadrant, the representative keywords included “Mice,” “Antipoxvirus compound st-246,” and “virulence.” The third quadrant is emerging or declining themes (lower values of centrality and density), which are not fully developed or marginally interesting for the domain. The fourth quadrant is niche themes (lower values of centrality and higher values of density), which have strongly developed but are still marginal for the domain under investigation. These keywords, including “Cynomolgus macaques,” “to-human transmission,” “adverse events,” and “immunogenicity,” belong to the two themes.

## Citations

### Which publications have cited these 100 studies?

A total of 2866 publications cited the top 100 most-cited studies. The 2866 publications were produced by 11 681 authors from 3211 institutions and 119 countries. Table 3 revealed the top 20 authors, institutions, and countries.

**Table 3:** The top 20 authors, institutions, and countries in the citing articles

Rank	Authors	Articles	Institutions	Articles	Countries	Articles
1	IK, Damon	82	Ctr Dis Control & Prevent	187	USA	1520
2	GR, Mary	58	NIAID	115	Germany	217
3	SC, Darin	43	University of St Augustine	83	England	203
4	AO, Victoria	40	Katholieke University Leuven	70	India	184
5	Y, Li	39	University of Pennsylvania	69	People’s Republic of China	138
6	LK, Kevin	38	Saint Louis University	60	France	133
7	DC, Erik	33	Emory University	57	Canada	122
8	MM, Andrea	33	Universidade Federal de Minas Gerais	46	Belgium	97
9	EH, Dennis	29	University of Florida	45	Russia	95
10	N, Andreas	25	FDA	45	Brazil	90
11	PB, Jahrling	24	NIH	43	Australia	73
12	GK, Erna	24	Robert Koch Institute	43	Spain	73
13	M, Bernard	24	Stanford University	40	Italy	71
14	J, Robert	23	Oregon Health and Science University	39	Japan	61
15	C, Shane	22	The University of Alabama at Birmingham	39	Nigeria	57
16	P, Scott	21	State Res Ctr Virol & Biotechnol Vector	38	Switzerland	55
17	A, Graciela	20	Harvard University	36	Democratic Republic of the Congo	47
18	MH, Christine	20	University of California San Diego	34	The Netherlands	42
19	LH, Christina	19	Russian Academy of Sciences	32	South Korea	42
20	N, Yoshinori	19	University of California	32	Egypt	37

**Figure 4:**
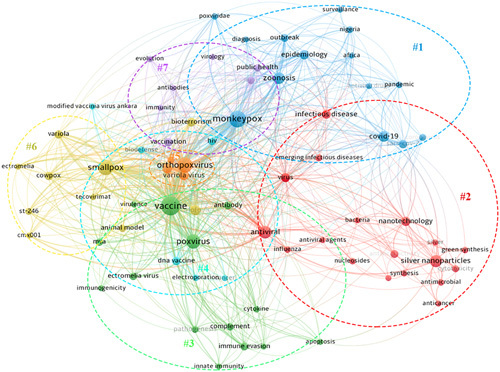
Keyword clustering analysis of the 2866 publications cited the top 100 studies.

We used VOSviewer (Fig. 4) to analyze the keywords and formed seven clusters, different clusters represent different research directions. Cluster 1 to cluster 7 represent infectious diseases, research on therapeutic drugs, vaccine development, animal models, poxvirus, antiviral drugs, and cellular immunity, respectively.

### The references of these 100 studies

A total of 1143 references were cited in the top 100 studies. Table 4 revealed the top 23 co-cited references (number of citations >10). The most co-cited study was published by Likos and colleagues (*n*=27) in 2005, followed by studies published by Reed and colleagues (*n*=23) in 2005 and Chen and colleagues (*n*=20) in 2005. A total of 813 co-cited authors in the 1143 references. Table 5 revealed the top 11 co-cited authors (number of citations ≥20). Jezek from the USA/Switzerland was the most co-cited author (*n*=63), followed by Reynolds (*n*=48) and Hutson (*n*=47) from the USA.

**Table 4:** Top 23 co-cited references

Rank	Co-cited reference	Co-citations
1	Likos AM, 2005, *J Gen Virol*, v86, p2661, doi 10.1099/vir.0.81215-0	27
2	Reed KD, 2004, *New Engl J Med*, v350, p342, doi 10.1056/nejmoa032299	23
3	Chen NH, 2005, *Virology*, v340, p46, doi 10.1016/j.virol.2005.05.030	20
4	Rimoin AW, 2010, p *Natl Acad Sci USA*, v107, p16262, doi 10.1073/pnas.1005769107	19
5	Hutinyjf, 2001, *Emerg Infect Dis*, v7, p434	18
6	Learned LA, 2005, *Am J Trop Med Hyg*, v73, p428, doi 10.4269/ajtmh.2005.73.428	18
7	Breman JG, 1980^9^, *B World Health Organ*, v58, p165	15
8	Hutson CL, 2009, *J Gen Virol*, v90, p323, doi 10.1099/vir.0.005108-0	15
9	Reynolds MG, 2006, *J Infect Dis*, v194, p773, doi 10.1086/505880	15
10	Zaucha GM, 2001, *Lab Invest*, v81, p1581, doi 10.1038/labinvest.3780373	15
11	Ladnyj ID, 1972^6^, *B World Health Organ*, v46, p593	14
12	Fine PEM, 1988, *Int J Epidemiol*, v17, p643, doi 10.1093/ije/17.3.643	13
13	Parker S, 2007, *Future Microbiol*, v2, p17, doi 10.2217/17460913.2.1.17	13
14	Earl PL, 2004, *Nature*, v428, p182, doi 10.1038/nature02331	12
15	Fenner F, 1988, *Smallpox Its Eradica*, doi 10.1136/jech.43.1.92	12
16	Hutson CL, 2007, *Am J Trop Med Hyg*, v76, p757, doi 10.4269/ajtmh.2007.76.757	12
17	Meyer H, 2002, *J Clin Microbiol*, v40, p2919, doi 10.1128/jcm.40.8.2919-2921.2002	12
18	Teshrb, 2004, *Emerg Infect Dis*, v10, p1563, doi 10.3201/eid1009.040310	12
19	Di Giulio DB, 2004, *Lancet Infect Dis*, v4, p15, doi 10.1016/s1473-3099(03)00856-9	11
20	Huhngd, 2005, *Clin Infect Dis*, v41, p1742, doi 10.1086/498115	11
21	Jezek Z, 1987, *J Infect Dis*, v156, p293, doi 10.1093/infdis/156.2.293	11
22	Li Y, 2006, *J Clin Virol*, v36, p194, doi 10.1016/j.jcv.2006.03.012	11
23	Xiao SY, 2005, *Emerg Infect Dis*, v11, p539, doi 10.3201/eid1104.040907	11

**Table 5:** Top 11 co-cited authors

Rank	Co-author	Institution	Country	Citations
1	Z, Jezek	World Health Organization	USA/Switzerland	63
2	MG, Reynolds	Centers for Disease Control and Prevention	USA	48
3	CL, Hutson	Centers for Disease Control and Prevention	USA	47
4	L, Khodakevich	World Health Organization	USA/Switzerland	39
5	AW, Rimoin	University of California	USA	30
6	JG, Breman^9^	National Institutes of Health	USA	36
7	PL, Earl	National Institutes of Allergy and Infectious Diseases	USA	30
8	AM, Likos	Centers for Disease Control and Prevention	USA	27
9	S, Parker	Arcturus Therapeutics, Inc.	USA	23
10	KD, Reed	Marshfield Clinic	USA	23
11	NH, Chen	State Key Laboratory of Bioactive Substances and Functions of Natural Medicines	China	20

**Figure 5:**
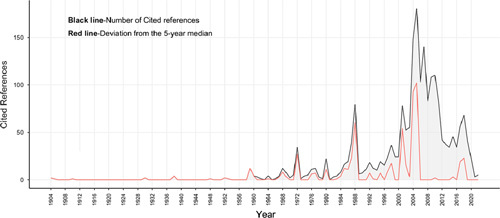
The reference publication year spectroscopy.

Figure 5 was the reference publication year spectroscopy, which is a quantitative method for identifying the historical origins of research fields and topics. We identified several time points that were important in the field of monkeypox research, including the years 1972, 1988, 2002, 2005, 2007, 2010, and 2018.

### 4 structural variation analysis

**Figure 6:**
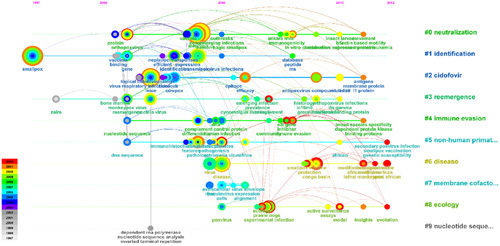
A timeline visualization of the top 100 most-cited papers published in 1997–2012.

**Figure 7:**
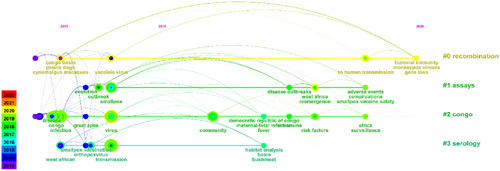
A timeline visualization of the top 100 most-cited papers published in 2013–2022.

Based on the annual publication of the top 100 most-cited papers in monkeypox (Fig. 1) and the results of the reference publication year spectroscopy (Fig. 5), we can see that the researches on monkeypox can be divided into two time periods, 1997–2012 and 2013–2022. In order to better identify promising and influential papers, this study conducted a structural variation analysis of the papers on monkeypox in these two time regions. The results were shown in Figures 6 and 7. The colored curves represent co-citation links added in the year of the corresponding color. Large-sized nodes or nodes with red treerings are of particular interest because they are either highly cited, or have citation bursts, or both^22^.

As we can see from Figure 6, in the time period 1997–2012, the larger nodes included smallpox, Congo, virus infection, etc. Cluster #6 was the key and hot spot for research, mainly including the monkeypox in West African, the monkeypox in Congo, and the lethal monkeypox. In Figure 7, we found that viruses, infections, and Congo were larger nodes, and the virus has always been a key point of research. In addition, MPXV, human-to-human transmission, vaccinia, and the epidemic of monkeypox in South Africa and Congo have been key research points in recent years.

## Discussion

The cited frequency represents the impact and influence of an article^8,23^. By doing a citation analysis of the top 100 most-cited articles in a certain research field, we can quantitatively determine the primary research focus, hot-topics, and future research trends^24^. Additionally, we can also determine that articles published across the journals exerted the most influence in the field^25^.

This study was the first bibliometric analysis of the top 100 most-cited articles within the field of monkeypox. We found that most of the top 100 most-cited articles were published between 2002 and 2011. The relevant articles on monkeypox have been published throughout the decades. Similarly, the studies on the poxvirus spanned across various decades. Therefore, our study provided important details about the trend in monkeypox research and development. In addition, the article type was also analyzed. We noted that the 100 top most-cited articles included 91 original studies and nine review articles, and about half of the original studies were mainly human experiment on the treatment and detection of monkeypox. There were also 39 original studies belonging to animal experimental studies, which mainly discussed the pathogenesis of monkeypox and vaccine development. Although only nine review articles were included in the analysis, we found that the mean citations per review was higher than that of the original article, and the median of annual citations were also higher than that of the original article. It may be that review articles are often more easily cited due to their comprehensive nature^26^.

We identified 711 different authors from 195 institutions and 28 countries among the top 100 most-cited articles. Damon from the USA Centers for Disease Control and Prevention published the highest number of papers (*n*=27), and he had the highest correlation with other authors. Similarly, 79% of the articles were from the United States, with an *H*-index of 49. For the analysis of the institutions, we found that the Centers for Disease Control and Prevention in the USA published most articles (*n*=34), followed by the University of St Augustine (*n*=19) and NIAID (*n*=12). The top three institutions in the published articles were all from the USA, one of which is a university, and the other two belong to the national public health management system. These findings indicated that the USA has made a significant contribution to the study of monkeypox. The high enthusiasm for academic activities in the USA has been attributed to the fact that its funding level for academic work is significantly higher than that of other countries, resulting in higher research quality^27^.

Our analysis indicated that the majority of the top 100 most-cited articles were published in the *New England Journal of Medicine*, followed by *Nanoscale Research Letters* and *Nature*. This may be due to the high quality of articles in these journals, or the authors tend to cite articles published in journals with high-impact factors^28^. The top 100 most-cited articles were published in 44 journals, and the articles from the last 5 years were published in 11 different journals. This discovery might reflect the rising research enthusiasm for monkeypox and also reflect the increasing number of monkeypox cases in 2022, which has attracted worldwide attention.

In bibliometrics, frequently occurring keywords can help to discover current research topics and hotspots and have a certain guiding effect on the development of scientific research^29,30^. We can see from the thematic map based on basic keywords that the research hot-topics of the top 100 most-cited articles were mainly about the infection and outbreak of monkeypox, and the basic research on the treatment of monkeypox, including vaccine evaluation, cross-protection, immunogenicity, and pathogenicity of the orthopoxvirus. Meanwhile, from the categories of the top 100 most-cited studies, we found that about one-third of the studies belonged to Immunology and one-third belonged to Infectious Diseases. This may be determined by the nature of monkeypox. From this, it can be seen that future research on this infectious disease mainly focuses on the immunological research field, with a forward-looking layout from basic research to translational medicine, providing technical reserves for future monkeypox epidemic prevention and control. After analyzing the 2866 documents that were cited by the top 100 most-cited studies on monkeypox, we found that these studies mainly focused on the public health security problems and coping strategies caused by the outbreak of orthopoxvirus, such as monkeypox, and also carried out research related to vaccines and therapeutic drugs. Additionally, our analysis of the cited references in the 100 studies found that the sudden increase in the number of studies in the field of monkeypox was distributed over 7 different years, which was closely related to the outbreak of monkeypox, prevention, and public health management.

The peak changes in monkeypox researches mainly occurred in two time periods: 1997–2012 and 2013–2022. Through structural variation analysis, we can see that the main research key points in the top 100 most-cited articles on monkeypox were concentrated in smallpox, the monkeypox epidemic in Congo and South Africa, the transmission route of monkeypox, the lethality of monkeypox, vaccines, etc., which was a progressive relationship in academic research. It was also the entire research process from the occurrence, understanding, and treatment of a disease. Therefore, it can be speculated that prevention and treatment will be the keys to future research on monkeypox. This also provided a management signal for health decision-makers to strengthen the prevention measures for monkeypox and increase the research and development efforts for monkeypox vaccines in order to tackle this infectious disease problem as soon as possible.

Since the outbreak of monkeypox in 2022, the WHO has immediately released temporary guidelines for monkeypox vaccines and immunization^31^. The guidelines pointed out that, based on the assessed risks and benefits at that time, regardless of the vaccine supply situation, large-scale smallpox vaccination is currently not needed or recommended for the prevention of monkeypox. In addition, the United States has authorized the treatment of monkeypox with drugs such as tecovimat and brincidofovir. Animal experiments have confirmed the efficacy of these two drugs on monkeypox, but there is still a lack of evidence for randomized clinical trials in patients with monkeypox. British researchers conducted a retrospective observational study^32^ and the results showed that the two antiviral drugs mentioned above may shorten the duration of monkeypox symptoms. There is an urgent need to conduct clinical randomized trials to evaluate the safety and efficacy of two drugs, not only in countries involved in the current monkeypox outbreak but also in areas where local epidemics occur. Developing second-line treatment drugs may be of great significance as the virus may develop resistance to first-line treatment drugs. This paper analyzed the top 100 most-cited articles on monkeypox by type of studies, countries, institutions, authors, journals, categories, keywords, etc., and provided the development status and hotpots of monkeypox research for scholars interested in this field. But our study still has several limitations: First, the data in this study is only from the WoSCC database and cannot completely cover the research of other databases, which may lead to the omission of research data. However, WoSCC is an authoritative and comprehensive database in the medical field. Second, this citation analysis did not exclude the influence of self-citations, which can inflate citation numbers for certain authors and journals. Third, citation counts can be influenced by factors other than impact, such as controversy or author reputation. Fourth, although the results have been carefully checked as guidelines^33^, there is still the possibility that the author has the same name and keywords have different expressions. Such errors can only be reduced, not completely avoided. Finally, there is a clear effect of time on the number of citation an articles receives. Due to the relatively short publication time of some included literatures, the number of citations was relatively small, which may bring a certain bias to the co-occurrence analysis results^34^.

## Conclusion

Overall, this study provided a detailed bibliometric analysis of the top 100 most-cited articles in monkeypox. Most studies focused on the infection and basic research on monkeypox. This bibliometric analysis of the top 100 most-cited articles in monkeypox can provide research hotspots and directions for researchers in this field. They can collaborate with countries that have contributed the most to monkeypox research in the world and conduct in-depth research on this disease, laying the foundation for better management of this disease in the future.

## Ethical approval

Ethics approval was not required for this review.

## Consent

Informed consent was not required for this review.

## Sources of funding

This study was supported by the Chinese Peking Union Medical College-Basic scientific research project of Central University (NO:2019XK320078, BJ-2019-092), Sailing project of Beijing Hospital Clinical Research in 2022 (BJ-2022-155).

## Author contributions

L.Z. and Y.Y.: methodology, software, and writing the manuscript. X.J.M. and Y.W.: planned and designed the study. L.Z. and K.H.Z. searched literatures and analyzed the data. All authors have read and approved the content of the manuscript.

## Conflicts of interest disclosure

The authors have no conflicts of interest to declare.

## Research registration unique identifying number (UIN)

This study does not include patients and does not belong to human studies.

## Guarantor

The guarantor of this study can be identified as Li Zheng, the first author of this article.

## Data availability statement

The original contributions presented in the study are included in the article or supplementary material; further inquiries can be directed to the corresponding authors.

## Provenance and peer review

This study was not invited.

## Supplementary Material
