# Supplementary material for: The top 100 most-cited studies on monkeypox: a brief bibliometric analysis
Source: Ann Med Surg (Lond). 2023 Oct 2;85(11):5568–76. doi: 10.1097/MS9.0000000000001367 (PMC10617903; doi:10.1097/MS9.0000000000001367)
Supplement: Supplementary file 1 [file ms9-85-5568-s001.docx]

**Table S1.** The top 100 most cited studies on monkeypox

| **Rank** | **Studies (first author, title, journal and year)** | **Citations** | |
| --- | --- | --- | --- |
| 1 | Reed, KD. The detection of monkeypox in humans in the Western Hemisphere. New England Journal of Medicine. 2004. | 382 |  |
| 2 | Rogers, James V. A preliminary assessment of silver nanoparticle inhibition of monkeypox virus plaque formation. Nanoscale Research Letters. 2008. | 270 |  |
| 3 | Earl, PL. Immunogenicity of a highly attenuated MVA smallpox vaccine and protection against monkeypox. Nature. 2004. | 267 |  |
| 4 | Rimoin, Anne W. Major increase in human monkeypox incidence 30 years after smallpox vaccination campaigns cease in the Democratic Republic of Congo. Proceedings of the National Academy of Sciences of the United States of America. 2010. | 230 |  |
| 5 | Edghill-Smith, Y. Smallpox vaccine-induced antibodies are necessary and sufficient for protection against monkeypox virus. Nature Medicine. 2005. | 210 |  |
| 6 | Di Giulio, DB. Human monkeypox: an emerging zoonosis. Lancet Infectious Diseases. 2004. | 204 |  |
| 7 | Likos, AM. A tale of two clades: monkeypox viruses. Journal of General Virology. 2005. | 201 |  |
| 8 | Hutin, YJF. Outbreak of human monkeypox, Democratic Republic of Congo, 1996-1997. Emerging Infectious Diseases. 2001. | 197 |  |
| 9 | Hooper, JW. Smallpox DNA vaccine protects nonhuman primates against lethal monkeypox. Journal of Virology. 2004. | 175 |  |
| 10 | De Clercq, E. Cidofovir in the treatment of poxvirus infections. Antiviral Research. 2002. | 166 |  |
| 11 | Chen, NH. Virulence differences between monkeypox virus isolates from West Africa and the Congo basin. Virology. 2005. | 142 |  |
| 12 | Baker, R. Potential antiviral therapeutics for smallpox, monkeypox and other orthopoxvirus infections. Antiviral Research. 2003. | 135 |  |
| 13 | Zaucha, GM. the pathology of experimental aerosolized monkeypox virus infection in cynomolgus monkeys (Macaca fascicularis). Laboratory Investigation. 2001. | 133 |  |
| 14 | Parker, Scott. Human monkeypox: an emerging zoonotic disease. Future Microbiology. 2007. | 127 |  |
| 15 | Stittelaar, KJ. Modified vaccinia virus Ankara protects macaques against respiratory challenge with monkeypox virus. Journal of Virology. 2005. | 127 |  |
| 16 | McCollum, Andrea M. Human Monkeypox. Clinical Infectious Diseases. 2014. | 122 |  |
| 17 | Learned, LA. Extended interhuman transmission of monkeypox in a hospital community in the Republic of the Congo, 2003. American Journal of Tropical Medicine and Hygiene. 2005. | 117 |  |
| 18 | Stittelaar, KJ. Antiviral treatment is more effective than smallpox vaccination upon lethal monkeypox virus infection. Nature. 2006. | 110 |  |
| 19 | Huhn, GD. Clinical characteristics of human monkeypox, and risk factors for severe disease. Clinical Infectious Diseases. 2005. | 109 |  |
| 20 | Heymann, DL. Re-emergence of monkeypox in Africa: a review of the past six years. British Medical Bulletin. 1998. | 108 |  |
| 21 | Meyer, H. Outbreaks of disease suspected of being due to human monkeypox virus infection in the Democratic Republic of Congo in 2001. Journal of Clinical Microbiology. 2002. | 103 |  |
| 22 | Guarner, J. Monkeypox transmission and pathogenesis in prairie dogs. Emerging Infectious Diseases. 2004. | 98 |  |
| 23 | Shchelkunov, SN. Human monkeypox and smallpox viruses: genomic comparison. Febs Letters. 2001. | 97 |  |
| 24 | Reynolds, Mary G. Clinical manifestations of human monkeypox influenced by route of infection. Journal of Infectious Diseases. 2006. | 93 |  |
| 25 | Durski, Kara N. Emergence of Monkeypox - West and Central Africa, 1970-2017. Mmwr-Morbidity and Mortality Weekly Report. 2018. | 91 |  |
| 26 | Hutson, Christina L. Monkeypox zoonotic associations: Insights from laboratory evaluation of animals associated with the multi-state us outbreak. American Journal of Tropical Medicine and Hygiene. 2007. | 88 |  |
| 27 | Hammarlund, E. Multiple diagnostic techniques identify previously vaccinated individuals with protective immunity against monkeypox. Nature Medicine. 2005. | 87 |  |
| 28 | Li, Yu. Detection of monkeypox virus with real-time PCR assays. Journal of Clinical Virology. 2006. | 86 |  |
| 29 | Huggins, John. Nonhuman Primates Are Protected from Smallpox Virus or Monkeypox Virus Challenges by the Antiviral Drug ST-246. Antimicrobial Agents and Chemotherapy. 2009. | 84 |  |
| 30 | Heraud, Jean-Michel. Subunit recombinant vaccine protects against monkeypox. Journal of Immunology. 2006. | 84 |  |
| 31 | Nalca, A. Reemergence of monkeypox: Prevalence, diagnostics, and countermeasures. Clinical Infectious Diseases. 2005. | 80 |  |
| 32 | Smee, DF. Characterization of wild-type and cidofovir-resistant strains of camelpox, cowpox, monkeypox, and vaccinia viruses. Antimicrobial Agents and Chemotherapy. 2002. | 80 |  |
| 33 | Vaughan, Aisling. Two cases of monkeypox imported to the United Kingdom, September 2018. Eurosurveillance. 2018. | 78 |  |
| 34 | Karem, KL. Characterization of acute-phase humoral immunity to monkeypox: Use of immunoglobulin M enzyme-linked immunosorbent assay for detection of monkeypox infection during the 2003 North American outbreak. Clinical and Diagnostic Laboratory Immunology. 2005. | 73 |  |
| 35 | Jin, YH. Practical synthesis of D- and L-2-cyclopentenone and their utility for the synthesis of carbocyclic antiviral nucleosides against orthopox viruses (smallpox, monkeypox, and cowpox virus). Journal of Organic Chemistry. 2003. | 73 |  |
| 36 | Sejvar, JJ. Human monkeypox infection: A family cluster in the Midwestern United States. Journal of Infectious Diseases. 2004. | 72 |  |
| 37 | Yinka-Ogunleye, Adesola. Outbreak of human monkeypox in Nigeria in 2017-18: a clinical and epidemiological report. Lancet Infectious Diseases. 2019. | 71 |  |
| 38 | Hutson, Christina L. A prairie dog animal model of systemic orthopoxvirus disease using West African and Congo Basin strains of monkeypox virus. Journal of General Virology. 2009. | 71 |  |
| 39 | Rimoin, Anne W. Endemic human monkeypox, democratic Republic of Congo, 2001-2004. Emerging Infectious Diseases. 2007. | 71 |  |
| 40 | Shchelkunov, SN. Analysis of the monkeypox virus genome. Virology. 2002. | 71 |  |
| 41 | Liszewski, M. Kathryn. Structure and regulatory profile of the monkeypox inhibitor of complement: Comparison to homologs in vaccinia and variola and evidence for dimer formation. Journal of Immunology. 2006. | 70 |  |
| 42 | Earl, Patricia L. Rapid protection in a monkeypox model by a single injection of a replication-deficient vaccinia virus. Proceedings of the National Academy of Sciences of the United States of America. 2008. | 66 |  |
| 43 | Levine, Rebecca S. Ecological Niche and Geographic Distribution of Human Monkeypox in Africa. Plos One. 2007. | 65 |  |
| 44 | Sklenovska, Nikola. Emergence of Monkeypox as the Most Important Orthopoxvirus Infection in Humans. Frontiers in Public Health. 2018. | 64 |  |
| 45 | Mukinda, VBK. Re-emergence of human monkeypox in Zaire in 1996. Lancet. 1997. | 64 |  |
| 46 | Reynolds, Mary G. Outbreaks of human monkeypox after cessation of smallpox vaccination. Trends in Microbiology. 2012. | 63 |  |
| 47 | Jordan, Robert. ST-246 Antiviral Efficacy in a Nonhuman Primate Monkeypox Model: Determination of the Minimal Effective Dose and Human Dose Justification. Antimicrobial Agents and Chemotherapy. 2009. | 62 |  |
| 48 | Beer, Ellen M. A systematic review of the epidemiology of human monkeypox outbreaks and implications for outbreak strategy. Plos Neglected Tropical Diseases. 2019. | 61 |  |
| 49 | Saijo, Masayuki. LC16m8, a highly attenuated vaccinia virus vaccine lacking expression of the membrane protein B5R, protects monkeys from monkeypox. Journal of Virology. 2006. | 59 |  |
| 50 | Erez, Noam. Diagnosis of Imported Monkeypox, Israel, 2018. Emerging Infectious Diseases. 2019. | 58 |  |
| 51 | Tesh, RB. Experimental infection of ground squirrels (Spermophiliustridecemlineatus) with Monkeypox virus. Emerging Infectious Diseases. 2004. | 58 |  |
| 52 | Hirao, Lauren A. Multival+B39ent Smallpox DNA Vaccine Delivered by Intradermal Electroporation Drives Protective Immunity in Nonhuman Primates Against Lethal Monkeypox Challenge. Journal of Infectious Diseases. 2011. | 56 |  |
| 53 | Reeves, Patrick M. Variola and Monkeypox Viruses Utilize Conserved Mechanisms of Virion Motility and Release That Depend on Abl and Src Family Tyrosine Kinases. Journal of Virology. 2011. | 56 |  |
| 54 | Damon, Inger K. Status of human monkeypox: clinical disease, epidemiology and research. Vaccine. 2011. | 53 |  |
| 55 | Xiao, SY. Experimental infection of prairie dogs with monkeypox virus. Emerging Infectious Diseases. 2005. | 53 |  |
| 56 | Edghill-Smith, Y. Smallpox vaccine does not protect Macaques with AIDS from a lethal Monkeypox virus challenge. Journal of Infectious Diseases. 2005. | 53 |  |
| 57 | Stabenow, Jennifer. A Mouse Model of Lethal Infection for Evaluating Prophylactics and therapeutics against Monkeypox Virus. Journal of Virology. 2010. | 51 |  |
| 58 | Nolen, Leisha Diane. Extended Human-to-Human Transmission during a Monkeypox Outbreak in the Democratic Republic of the Congo. Emerging Infectious Diseases. 2016. | 50 |  |
| 59 | Formenty, Pierre. Human Monkeypox Outbreak Caused by Novel Virus Belonging to Congo Basin Clade, Sudan, 2005. Emerging Infectious Diseases. 2010. | 50 |  |
| 60 | Yinka-Ogunleye, Adesola. Reemergence of Human Monkeypox in Nigeria, 2017. Emerging Infectious Diseases. 2018. | 47 |  |
| 61 | Radonic, Aleksandar. Fatal Monkeypox in Wild-Living Sooty Mangabey, Cole d'Ivoire, 2012. Emerging Infectious Diseases. 2014. | 46 |  |
| 62 | Americo, Jeffrey L. Identification of Wild-Derived Inbred Mouse Strains Highly Susceptible to Monkeypox Virus Infection for Use as Small Animal Models. Journal of Virology. 2010. | 45 |  |
| 63 | Manes, Nathan P. Comparative proteomics of human monkeypox and vaccinia intracellular mature and extracellular enveloped virions. Journal of Proteome Research. 2008. | 45 |  |
| 64 | Petersen, Eskild. Human Monkeypox Epidemiologic and Clinical Characteristics, Diagnosis, and Prevention. Infectious Disease Clinics of North America. 2019. | 42 |  |
| 65 | Reynolds, Mary G. Spectrum of infection and risk factors for human monkeypox, United States, 2003. Emerging Infectious Diseases. 2007. | 42 |  |
| 66 | Thornhill, John P. Monkeypox Virus Infection in Humans across 16 Countries - April-June 2022. New England Journal of Medicine. 2022. | 41 |  |
| 67 | Vaughan, Aisling. Human-to-Human Transmission of Monkeypox Virus, United Kingdom, October 2018. Emerging Infectious Diseases. 2020. | 40 |  |
| 68 | Kugelman, Jeffrey R. Genomic Variability of Monkeypox Virus among Humans, Democratic Republic of the Congo. Emerging Infectious Diseases. 2014. | 40 |  |
| 69 | Parker, Scott. A review of experimental and natural infections of animals with monkeypox virus between 1958 and 2012. Future Virology. 2013. | 40 |  |
| 70 | Li, Yu; Zhao, Hui. Real-time PCR assays for the specific detection of monkeypox virus West African and Congo Basin strain DNA. Journal of Virological Methods. 2010. | 40 |  |
| 71 | Sbrana, Elena. Comparative pathology of North American and central African strains of monkeypox virus in a ground squirrel model of the disease. American Journal of Tropical Medicine and Hygiene. 2007. | 40 |  |
| 72 | Antinori, Andrea. Epidemiological, clinical and virological characteristics of four cases of monkeypox support transmission through sexual contact, Italy, May 2022. Eurosurveillance. 2022. | 39 |  |
| 73 | Doty, Jeffrey B. Assessing Monkeypox Virus Prevalence in Small Mammals at the Human-Animal Interface in the Democratic Republic of the Congo. Viruses-Basel. 2017. | 38 |  |
| 74 | Goff, Arthur J. A Novel Respiratory Model of Infection with Monkeypox Virus in Cynomolgus Macaques. Journal of Virology. 2011. | 38 |  |
| 75 | Nalca, Aysegul. Experimental Infection of Cynomolgus Macaques (Macaca fascicularis) with Aerosolized Monkeypox Virus. Plos One. 2010. | 38 |  |
| 76 | Osorio, Jorge E. Comparison of Monkeypox Viruses Pathogenesis in Mice by In Vivo Imaging. Plos One. 2009. | 38 |  |
| 77 | Karem, Kevin L. Monkeypox-induced immunity and failure of childhood smallpox vaccination to provide complete protection. Clinical and Vaccine Immunology. 2007. | 38 |  |
| 78 | Reynolds, Mary G. Factors affecting the likelihood of monkeypox's emergence and spread in the post-smallpox era. Current Opinion in Virology. 2012. | 37 |  |
| 79 | Smith, Scott K. Effective Antiviral Treatment of Systemic Orthopoxvirus Disease: ST-246 Treatment of Prairie Dogs Infected with Monkeypox Virus. Journal of Virology. 2011. | 37 |  |
| 80 | Chu, CK. Antiviral activity of cyclopentenyl nucleosides against orthopox viruses (smallpox, monkeypox and cowpox). Bioorganic & Medicinal Chemistry Letters. 2003. | 37 |  |
| 81 | Alakunle, Emmanuel. Monkeypox Virus in Nigeria: Infection Biology, Epidemiology, and Evolution. Viruses-Basel. 2020. | 36 |  |
| 82 | Simpson, Karl. Human monkeypox - After 40 years, an unintended consequence of smallpox eradication. Vaccine. 2020. | 36 |  |
| 83 | Brainard, Julii. Misinformation making a disease outbreak worse: outcomes compared for influenza, monkeypox, and norovirus. Simulation-Transactions of the Society for Modeling and Simulation International. 2020. | 36 |  |
| 84 | Buchman, George W. A protein-based smallpox vaccine protects non-human primates from a lethal monkeypox virus challenge. Vaccine. 2010. | 36 |  |
| 85 | Smith, Scott K. In Vitro Efficacy of ST246 against Smallpox and Monkeypox. Antimicrobial Agents and Chemotherapy. 2009. | 36 |  |
| 86 | Schultz, Denise A. Experimental infection of an African dormouse (Graphiuruskelleni) with monkeypox virus. Virology. 2009. | 36 |  |
| 87 | Rubins, Kathleen H. Comparative Analysis of Viral Gene Expression Programs during Poxvirus Infection: A Transcriptional Map of the Vaccinia and Monkeypox Genomes. Plos One. 2008. | 36 |  |
| 88 | Lederman, Edith R. Prevalence of antibodies against orthopoxviruses among residents of Likouala region, Republic of Congo: Evidence for Monkeypox virus exposure. American Journal of Tropical Medicine and Hygiene. 2007. | 36 |  |
| 89 | Johnson, Reed F. Comparative Analysis of Monkeypox Virus Infection of Cynomolgus Macaques by the Intravenous or Intrabronchial Inoculation Route. Journal of Virology. 2011. | 35 |  |
| 90 | Weaver, Jessica R. Monkeypox virus and insights into its immunomodulatory proteins. Immunological Reviews. 2008. | 35 |  |
| 91 | Rubins, Kathleen H. Stunned Silence: Gene Expression Programs in Human Cells Infected with Monkeypox or Vaccinia Virus. Plos One. 2011. | 34 |  |
| 92 | Gileva, Irina P. Properties of the recombinant TNF-binding proteins from variola, monkeypox, and cowpox viruses are different. Biochimica Et Biophysica Acta-Proteins and Proteomics. 2006. | 34 |  |
| 93 | Kile, JC. Transmission of monkeypox among persons exposed to infected prairie dogs in Indiana in 2003. Archives of Pediatrics & Adolescent Medicine. 2005. | 33 |  |
| 94 | Fleischauer, AT. Evaluation of human-to-human transmission of monkeypox from infected patients to health care workers. Clinical Infectious Diseases. 2005. | 33 |  |
| 95 | Earl, Patricia L. Lethal Monkeypox Virus Infection of CAST/EiJ Mice Is Associated with a Deficient Gamma Interferon Response. Journal of Virology. 2012. | 32 |  |
| 96 | Saijo, Masayuki. Virulence and pathophysiology of the Congo Basin and West African strains of monkeypox virus in non-human primates. Journal of General Virology. 2009. | 32 |  |
| 97 | Rao, Agam K. Monkeypox in a Traveler Returning from Nigeria - Dallas, Texas, July 2021. Mmwr-Morbidity and Mortality Weekly Report. 2022. | 31 |  |
| 98 | Mbala, Placide K. Maternal and Fetal Outcomes Among Pregnant Women With Human Monkeypox Infection in the Democratic Republic of Congo. Journal of Infectious Diseases. 2017. | 31 |  |
| 99 | Hatch, Graham J. Assessment of the Protective Effect of Imvamune and Acam2000 Vaccines against Aerosolized Monkeypox Virus in Cynomolgus Macaques. Journal of Virology. 2013. | 31 |  |
| 100 | Hutson, Christina L. Dosage comparison of Congo Basin and West African strains of monkeypox virus using a prairie dog animal model of systemic orthopoxvirus disease. Virology. 2010. | 31 |  |

**Table S2.** Number of citations of the authors with more than 3 article

| **Rank** | **Author** | **Published studies** | **Total of Citations** | **Rank** | **Author** | **Published studies** | **Total of Citations** |
| --- | --- | --- | --- | --- | --- | --- | --- |
| 1 | IK, Damon | 27 | 2397 | 26 | HB, Zach | 3 | 183 |
| 2 | LK, Kevin | 9 | 431 | 27 | C, Jennifer | 3 | 184 |
| 3 | GR, Mary | 9 | 565 | 28 | F, Monika | 3 | 138 |
| 4 | LR, Russell | 7 | 908 | 29 | M, Eric | 3 | 184 |
| 5 | AO, Victoria | 6 | 368 | 30 | BW, Carly | 3 | 184 |
| 6 | JJ, Esposito | 6 | 772 | 31 | F, Pierre | 3 | 421 |
| 7 | PB, Jahrling | 6 | 885 | 32 | DW, Nathan | 3 | 341 |
| 8 | SC, Darin | 5 | 265 | 33 | LW, Linda | 3 | 341 |
| 9 | EH, Lisa | 5 | 402 | 34 | F, Pierre | 3 | 351 |
| 10 | WR, Anne | 5 | 408 | 35 | LK, Kevin | 3 | 139 |
| 11 | B, Zachary | 4 | 197 | 36 | NL, Vladimir | 3 | 333 |
| 12 | J, Robert | 4 | 219 | 37 | BD, Whitni | 3 | 185 |
| 13 | KS, Scott | 4 | 160 | 38 | JM, Benjamin | 3 | 435 |
| 14 | Y, Li | 4 | 772 | 39 | LA, Jeffrey | 3 | 143 |
| 15 | AO, Victoria | 4 | 772 | 40 | LE, Patricia | 3 | 143 |
| 16 | JK, Matthew | 4 | 247 | 41 | M, Bernard | 3 | 143 |
| 17 | GR, Mary | 4 | 525 | 42 | AN, Ysegul | 3 | 343 |
| 18 | MH, Christine | 4 | 202 | 43 | AW, Chris | 3 | 343 |
| 19 | SK, Abdul | 4 | 327 | 44 | WH, John | 3 | 482 |
| 20 | B, Mike | 4 | 478 | 45 | H, Jennifer | 3 | 163 |
| 21 | MM, Andrea | 4 | 301 | 46 | LB, Mark | 3 | 233 |
| 22 | LH, Christina | 3 | 190 | 47 | I, Chikwe | 3 | 125 |
| 23 | W, Sonja | 3 | 139 | 48 | P, Scott | 3 | 218 |
| 24 | EH, Dennis | 3 | 135 | 49 | S, Peter | 3 | 176 |
| 25 | TH, John | 3 | 186 |  |  |  |  |

**Table S3.** Citation (all, median) and H-index of monkeypox research in top 10 countries

| **Rank** | **Country** | **Published studies** | **H-index** | **Total of citation** | **Median of citation** |
| --- | --- | --- | --- | --- | --- |
| 1 | USA | 79 | 49 | 6467 | 81.86 |
| 2 | Switzerland | 12 | 12 | 1345 | 112.08 |
| 3 | Germany | 8 | 8 | 770 | 96.25 |
| 4 | Dem Rep Congo | 8 | 8 | 627 | 78.38 |
| 5 | England | 8 | 8 | 365 | 45.63 |
| 6 | Nigeria | 6 | 6 | 310 | 51.67 |
| 7 | Belgium | 5 | 5 | 601 | 120.20 |
| 8 | Netherlands | 3 | 3 | 279 | 93.00 |
| 9 | Rep Congo | 3 | 3 | 244 | 81.33 |
| 10 | Russia | 3 | 3 | 202 | 67.33 |

**Table S4.** Number of studies and citations of the top 10 institutions

| **Rank** | **Organization** | **Published studies** | **Total of citations** |
| --- | --- | --- | --- |
| 1 | Ctr Dis Control & Prevent | 34 | 2728 |
| 2 | University of St Augustine | 19 | 1988 |
| 3 | NIAID | 12 | 1228 |
| 4 | WHO | 10 | 1253 |
| 5 | Southern Research | 6 | 505 |
| 6 | Saint Louis University | 6 | 466 |
| 7 | US Ctr Dis Control & Prevent | 6 | 306 |
| 8 | Indiana State Health Department | 5 | 268 |
| 9 | Public Health England | 5 | 256 |
| 10 | University of California | 5 | 452 |

**Table S5.** Journals and numbers of published papers

| **Journal** | **Published studies** | **Citations** | **IF_2022_** |
| --- | --- | --- | --- |
| Emerging Infectious Diseases | 13 | 850 | 16.126 |
| Journal of Virology | 11 | 686 | 6.549 |
| Journal of Infectious Diseases | 5 | 305 | 7.759 |
| Plos One | 5 | 211 | 3.752 |
| American Journal of Tropical Medicine and Hygiene | 4 | 281 | 3.707 |
| Antimicrobial Agents and Chemotherapy | 4 | 262 | 5.938 |
| Clinical Infectious Diseases | 4 | 344 | 20.999 |
| Virology | 4 | 280 | 3.513 |
| Journal of General Virology | 3 | 304 | 5.141 |
| Vaccine | 3 | 125 | 4.169 |
| Antiviral Research | 2 | 301 | 10.103 |
| Eurosurveillance | 2 | 117 | 21.286 |
| Journal of Immunology | 2 | 154 | 5.426 |
| Lancet Infectious Diseases | 2 | 275 | 71.421 |
| MMWR-Morbidity and Mortality Weekly Report | 2 | 122 | 35.301 |
| Nature | 2 | 377 | 69.504 |
| Nature Medicine | 2 | 297 | 87.241 |
| New England Journal of Medicine | 2 | 423 | 176.079 |
| Proceedings of the National Academy of Sciences of the United States of America | 2 | 296 | 12.779 |
| Viruses-Basel | 2 | 74 | 5.818 |
| Archives of Pediatrics & Adolescent Medicine | 1 | 33 | 0 |
| Biochimica et Biophysica Acta-Proteins and Proteomics | 1 | 34 | 4.125 |
| Bioorganic & Medicinal Chemistry Letters | 1 | 37 | 2.94 |
| British Medical Bulletin | 1 | 108 | 5.841 |
| Clinical and Diagnostic Laboratory Immunology | 1 | 73 | 5.732 |
| Clinical and Vaccine Immunology | 1 | 38 | 0 |
| Current Opinion in Virology | 1 | 37 | 7.121 |
| Febs Letters | 1 | 97 | 3.864 |
| Frontiers in Public Health | 1 | 64 | 6.461 |
| Future Microbiology | 1 | 127 | 3.553 |
| Future Virology | 1 | 40 | 3.015 |
| Immunological Reviews | 1 | 35 | 10.983 |
| Infectious Disease Clinics of North America | 1 | 42 | 5.905 |
| Journal of Clinical Microbiology | 1 | 103 | 11.677 |
| Journal of Clinical Virology | 1 | 86 | 14.481 |
| Journal of Organic Chemistry | 1 | 73 | 4.198 |
| Journal of Proteome Research | 1 | 45 | 5.37 |
| Journal of Virological Methods | 1 | 40 | 2.623 |
| Laboratory Investigation | 1 | 133 | 5.502 |
| Lancet | 1 | 64 | 202.731 |
| Nanoscale Research Letters | 1 | 270 | 5.418 |
| PLOS Neglected Tropical Diseases | 1 | 61 | 4.781 |
| Simulation-Transactions of the Society for Modeling and Simulation International | 1 | 36 | 1.699 |
| Trends in Microbiology | 1 | 63 | 18.23 |

**Figure S1.** Cooperation network map of the top 50 authors


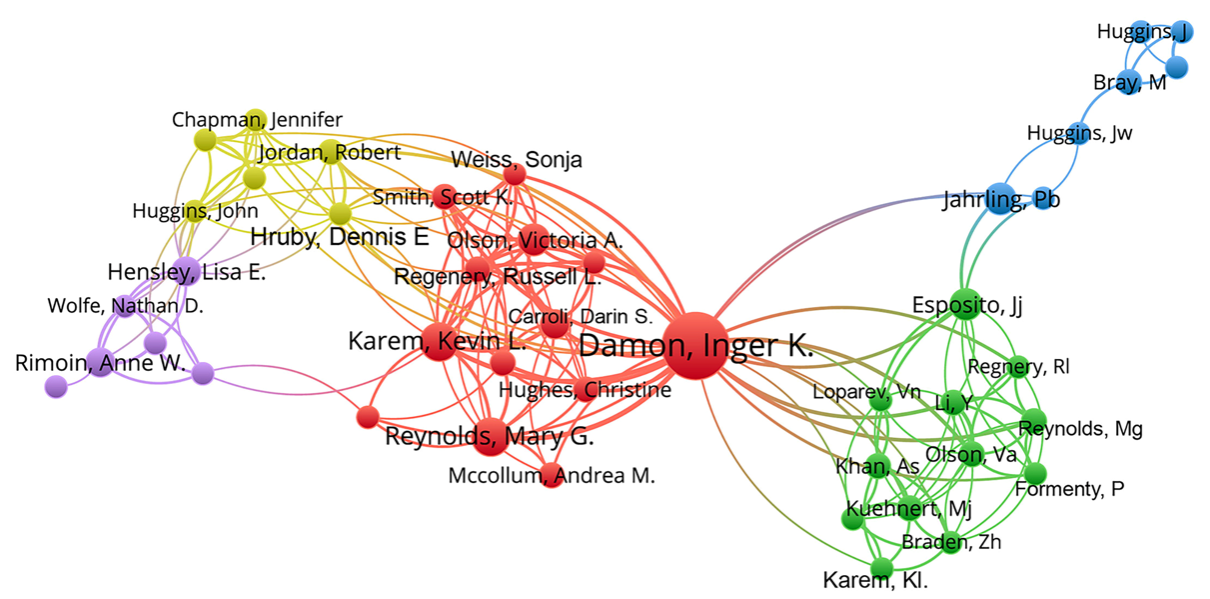


The node size represents the number of items and the lines between nodes reflect the collaboration or co-citation relationship of items. Centrality is an indicator used to measure the importance of an element. The closer to the center of the map, the stronger the centrality.

**Figure S2.**Cooperation network map of the countries


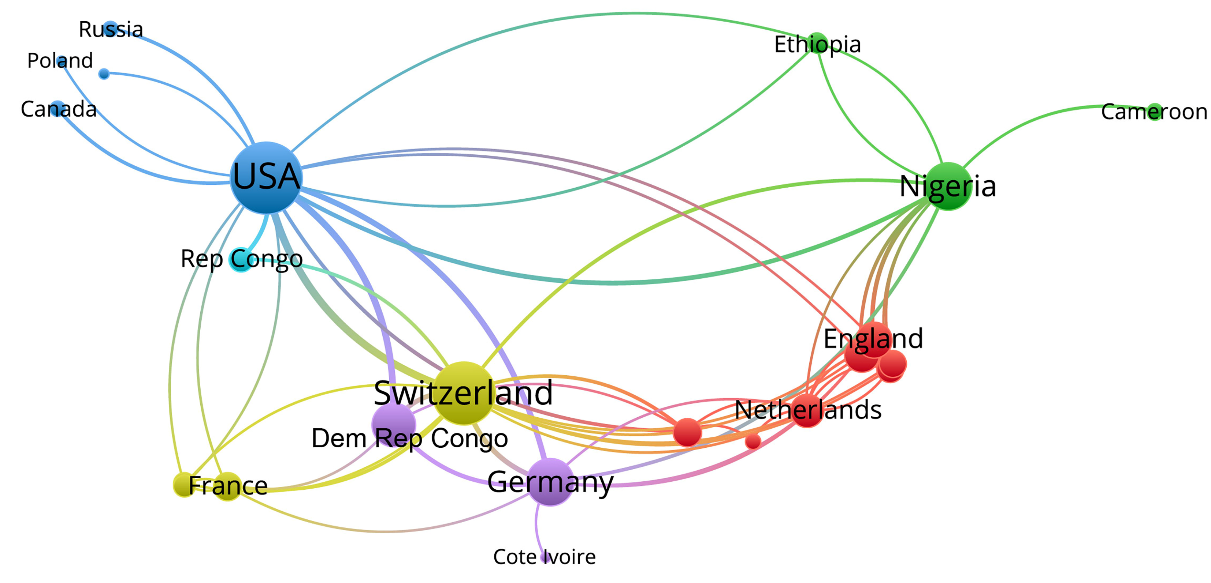


The node size represents the number of items and the lines between nodes reflect the collaboration or co-citation relationship of items. Centrality is an indicator used to measure the importance of an element. The closer to the center of the map, the stronger the centrality.

**Figure S3.** Cooperation network map of the institutions


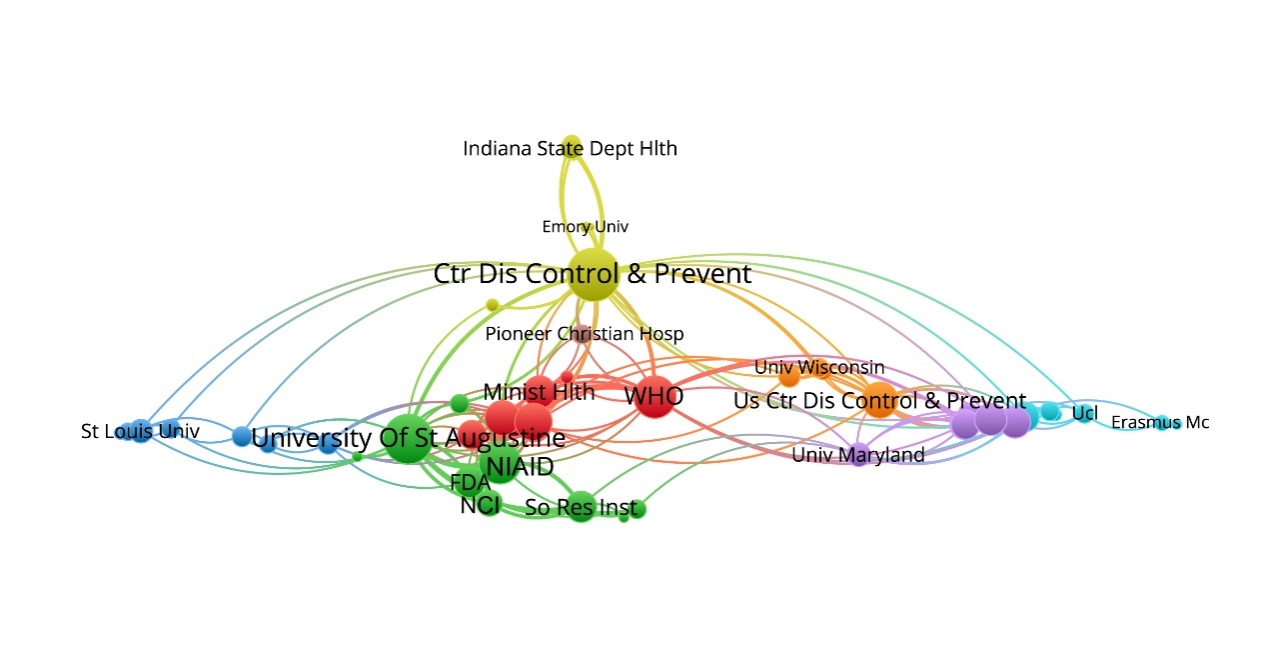


The node size represents the number of items and the lines between nodes reflect the collaboration or co-citation relationship of items. Centrality is an indicator used to measure the importance of an element. The closer to the center of the map, the stronger the centrality.

**Figure S4.**Number of papers issued by journals within the different IF range

**
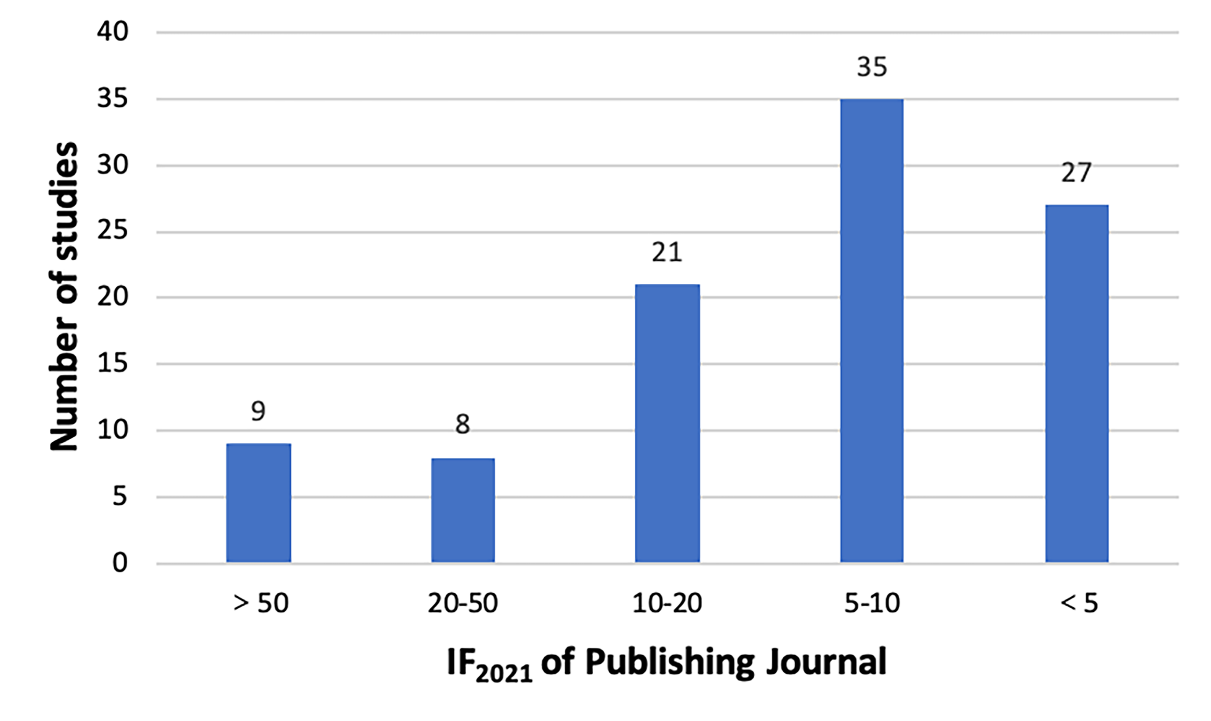
**
